# Mandibular advancement device: prescription in adult dental sleep medicine — guideline of the German Society of Dental Sleep Medicine

**DOI:** 10.1007/s11325-022-02601-6

**Published:** 2022-03-29

**Authors:** Olaf Bernhardt, Nikolaos Nikitas Giannakopoulos, Markus Heise, Alexander Meyer, Dagmar Norden, Jörg Schlieper, Horst Kares

**Affiliations:** 1 Department of Restorative Dentistry, Periodontology, Endodontology, Preventive Dentistry and Pediatric Dentistry, University Medicine Greifswald, W. Rathenaustr. 42 a, 17475 Greifswald, Germany; 2 Department of Prosthodontics, University Clinic of Würzburg, Pleicherwall 2, 97070 Würzburg, Germany; 3 Department of Prosthodontics, National and Kapodistrian University of Athens, Athens, Greece; 4 Private Practice, Alleestrasse 80, 44793 Bochum, Germany; 5 Private Practice, Friedrich-Ebert-Straße 21, 42719 Solingen, Germany; 6 Private Practice, Theaterwall 4, 26122 Oldenburg, Germany; 7 Private Practice, Osdorfer Weg 147, 22607 Hamburg, Germany; 8 Private Practice, Grumbachtalweg 9, 66121 Saarbrücken, Germany

**Keywords:** Obstructive sleep apnea, Dental sleep medicine, Mandibular advancement device, Guideline, Clinical procedure

## Abstract

**Purpose:**

Obstructive sleep apnea (OSA) may result in severe health onditions, reduces quality of live, and affects high percentages of the adult population. Due to recent changes in the German health care regulations, mandibular advancement devices (MAD) will become available as a treatment option for OSA to a greater extent for general dentists and their patients.

**Methods:**

A guideline development group consisting of nine members representing four German dental and medical organizations was formed, in order to provide critical information and orientation to the main stakeholders (dentists and patients), regarding the use of MAD for the treatment of OSA within dental sleep medicine.

**Results:**

This guideline aims to inform physicians and dentists, particularly those with acquired qualification/specialization in sleep medicine (or in the diagnosis and treatment of sleep-related breathing disorders), as well as experts, payers, and patients. It delivers recommendations on technical requirements for MAD prescription and fabrication, clinical procedures, maintenance, and follow-up procedures.

**Conclusion:**

A MAD should be designed for long-term therapy and must be a custom made, adjustable, bimaxillary retained two-splint system equipped with adjustable protrusive elements. The fabrication in a dental laboratory should be based on dental impressions or scans and three-dimensional registrations of the starting position taken with a bite gauge.

## Introduction

While initial studies reported the prevalence of obstructive sleep apnea (OSA) to be 4% of the male population and 2% of the female population in the USA [1], recent studies now reflect a much higher prevalence. A study in 2005 of the prevalence of OSA in the Swiss general population found that 49.7% of men and 23.4% of women aged 40 to 80 years had moderate to severe OSA, defined as a median Apnea–Hypopnea Index (AHI) score of more than 15 events per hour [2]. The prevalence was even higher when all severity grades (mild to severe OSA) were included: 83.8% in men and 60.8% in women. Even with the current criteria of the much more sensitive International Classification of Sleep Disorders, 3rd Edition (ICSD-3) [3], the prevalence of OSA was estimated to be 79.2% in men and 54.3% in women of the over-40 age group. In contrast, prevalence estimates for snoring vary widely from 2 to 86% due to the frequent lack of clear differentiation between snoring and OSA [4].

Treatment recommendations vary, depending on the severity of OSA and snoring, from general measures such as weight reduction, exercise or positional therapy, to more specific treatments such as positive airway pressure (PAP) therapy, occlusal splint therapy with a mandibular advancement device (MAD), and surgical procedures [5]. Compared to PAP therapy, oral appliance therapy (OAT) results in better adherence to treatment and, thus, to comparable effectiveness with fewer side effects compared to PAP therapy [6, 7].

MADs are used in adults for the management of snoring, OSA, and of the health and social impairments associated with these sleep disorders. Depending on the specific constellation of medical findings, mandibular advancement devices may be used alone or in combination with other treatment options such as diet for obesity, positional therapy for positional OSA and snoring, in combination with positive airway pressure therapy in order to lower the therapeutic PAP-pressure, as well as with surgical methods.

Any oral appliance used for MAD for treatment of OSA should be a custom-made, adjustable, bimaxillary retained, two-splint (bibloc) system with adjustable protrusive elements fabricated in a dental laboratory based on dental impressions or scans and three-dimensional registrations of the starting position taken with a bite gauge. The mechanism of action of MAD therapy is predominantly induced by mandibular protrusion, which places tension on the suprahyoid tissues, resulting in luminal enlargement and stabilization of the airway at the level of the velum, the tongue base, and the epiglottis [8]. Due to the entry into force of the decision by the Federal Joint Committee (G-BA) [9] to include the “mandibular advancement device” as a treatment option for OSA in “Guideline Methods of Contractual Medical Care” (MVV-RL) of February 24 2021, and due to the G-BA decision to amend the guideline entitled “Mandibular advancement device for obstructive sleep apnea” of May 6, 2021 on adequate, appropriate, and cost-efficient contractual dental care, the aim of this guideline is to support dentists applying mandibular advancement devices in the field of dental sleep medicine by providing critical information on structure, process, and outcome orientation to improve the quality of care. This guideline aims to inform physicians and dentists, particularly those with acquired qualification/specialization in sleep medicine (or in the diagnosis and treatment of sleep-related breathing disorders), as well as experts, payers, and patients.

## Material and methods

The guideline development group consisted of four experts representing the following organizations: German Dental Association, German Society of Craniomandibular Function and Disorders, Orofacial Pain Working Group of the German Pain Society, the German Association of Statutory Health Insurance Dentists, and five experts representing the German Society of Dental Sleep Medicine. The methodology used for the development of this guideline is based on the AWMF Guideline on Guideline Development [10] (*AWMF-Regelwerk Leitlinien*, Version 1.1 of 27 March 2013).

The literature search was primarily conducted in PubMed and was limited to the last 10 years using the following terms in combination: obstructive sleep apnea, oral appliance, mandibular repositioning device, mandibular advancement device, and guidelines. In addition, a hand search was performed by all guideline authors; this search also considered earlier publications. A total of 124 full-text publications were identified for the preparation of this guideline.

Recommendations were developed by a consensus of the representatively composed guideline development group and submitted to the boards of the participating professional societies for adoption. The recommendations were developed based on discussion and voting in a structured consensus-building process. Fourteen online group meetings were held. Decisions on the individual topics were made by circular resolution via email, and final consensus was reached on 1 June 2021. The recommendation grading scheme used in this guideline is shown in Table 1. The strength of consensus was classified as shown in Table 2.

**Table 1:** Recommendation grading scheme

Wording of recommendations FOR an intervention	Wording of recommendations AGAINST an intervention	Recommendation grade
“must” (German: “*soll*”)/ “we strongly recommend”	“must not” (German: “*soll nicht*”/ “we discourage”	Strong recommendation
“should” (German: “*sollte*”)/ “we recommend”	“should not” (German: “*sollte nicht*”)/ “we do not recommend”	Recommendation
“could” (German: “*kann*”)/ “could be considered”	“can be omitted” (German: “*kann verzichtet werden*”)/ “is optional”	Open recommendation

**Table 2:** Classification of the strength of consensus (AWMF)

Classification of the strength of consensus	
Strong consensus	Agreement of > 95% of the participants
Consensus	Agreement of > 75 to 95% of the participants
Majority agreement	Agreement of > 50% to 75% of the participants
No consensus	Agreement of < 50% of the participants

## Results

### Recommendations for dentists providing MAD therapy

**Fig. 1:**
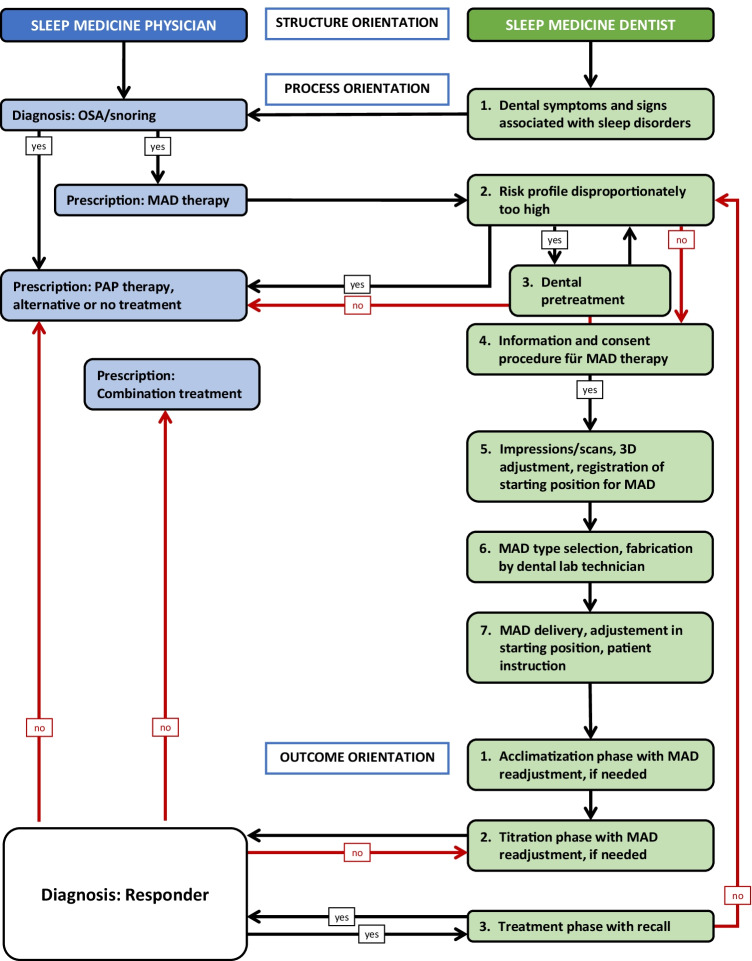
Clinical algorithm for mandibular advancement device (MAD

Prior to fabrication of a MAD a medical referral and prescription is legal requirement (Fig. 1). MAD providers should have a good knowledge about distinct oral appliances and their therapeutic applications. All skills and the underlying knowledge should be acquired or expanded through continuing education and training.

### Clinical procedure for dentists

#### Dental diagnosis and treatment

The aim of dental diagnostics and therapeutics before and during oral appliance therapy for sleep apnea and snoring is to optimize preconditions for treatment success and to minimize risks to the stomatognathic system.

Risks and benefits of MAD therapy have to be assessed from medical and dental perspectives using an interdisciplinary team approach. The dentist bears the sole responsibility to evaluate all risks associated with MAD use for each individual patient and, if necessary, to decide if dental pretreatment measures are needed before the start of OAT. Every risk assessment is based on the individual risk profile, i.e., the conservative, prosthetic, periodontal, and functional dental examination findings and constellations of findings relevant to MAD use (diagnostic indices and classifications including radiographic examinations) [4, 5, 11–14].

Clinical findings that may preclude a dental indication for MAD include:

- Insufficient stability of remaining teeth/insufficient occlusal support zones (increased risk, e.g., of Eichner Index Group B and C, disregarding teeth with mobility grades and insufficient prosthetic equators);
- Temporary removable of partial dentures (increased risk, e.g., in the presence of wire bended clasps) or permanent removable, fixed, and combined dentures (increased risk, e.g., in case of insufficient stability);
- Dental caries, insufficient fillings, or other defects (increased risk, e.g., in case of fillings with more than one surface are in need of repair);
- Periodontitis/periimplantitis (increased risk, e.g., in case of bleeding on probing [BOP] index > 10%, probing depth ≥ 4 mm, stage > 1, and grade > A) [15];
- Functional disorders of the masticatory system: increased risk in case of function-limiting and/or painful temporomandibular disorders (TMD), especially dysfunctional pain (grades 3 and 4 of the Graded Chronic Pain Scale) and function-limiting mandibular hypomobility in the presence of < 5 mm of mandibular protrusion, measured from the maximum retruded position as the best of three attempts, in accordance with the Diagnostic Criteria for Temporomandibular Disorders (DC/TMD) [16].

The patient’s individual profile of the aforementioned risk factors relevant to treatment decision-making may limit the indications and possibilities for MAD therapy, in the dentist’s assessment.

According to the above clinical findings, dentists determine whether and which dental measures need to be initiated before and during OAT for risk reduction, including a possible adjustment of a preexisting MAD.

Existing risk factor assessment tools which have been modified in order to capture the treatment-relevant findings and constellations of findings associated with OAT, e.g., vector diagram-type instruments used in dentistry, may be employed [14, 17]. If the patient requires a dental pretreatment that could potentially delay the initiation of MAD therapy, the referring physician should be informed (Table 3).

**Table 3:** Recommendations of the guideline development group: dental diagnosis and treatment

Recommendation 1	
We strongly recommend to make decisions regarding the indication for MAD therapy as a risk-based decision based on the dental findings and the constellations of findings Voting results: 8/0/0 (Yes/No/Abstain)	Strong consensus

#### Dental fabrication of a MAD

To ensure optimal fit and function of the oral appliance, precise impressions of the upper and lower arches form the basis for MAD fabrication. These may be conventional or digital impressions, according to the practitioner’s preferences. When taking the impressions, particular attention must be paid to adequate undercut and soft tissue rendering. Clinical parameters that can impair impression quality (hard or soft plaque, increased tooth mobility, etc.) must also be taken into account.

#### Vertical, sagittal, and horizontal dimension of the MAD position

The vertical dimension of the MAD starting position (longitudinal axis, bite elevation) remains controversial. There is no clear scientific evidence regarding the optimal vertical dimension and its effect on sleep-related breathing disorders. However, clinical experience has shown that the measured absolute protrusion of the mandible and adherence to MAD therapy decrease with increasing vertical dimension [18–21].

To prevent fracture of the device during treatment, the vertical distance in the posterior region should be large enough to enable adequate dental laboratory work on the respective MAD. The impact of a sharply developed curve of Spee on the interocclusal distance needs to be considered over the complete protrusive course of the mandible.

To prevent interference over the total/complete distance of mandibular protrusion, the vertical distance in the anterior region should be large enough to enable adequate dental laboratory work on the specific MAD, but not too large as to impede lip closure during sleep.

Recommendations regarding the sagittal dimension of the starting position of MAD therapy (retrusion/protrusion) vary greatly (from 6 to 90% of the maximum protrusion capacity) due to discrepancies in the reference point of measurement, among other things [22–25]. In contrast, literature consistently states that the usage of the active maximal retruded position as the reference point for measuring protrusive movement achieves superior reproducibility. Therefore, and for reasons of clinical feasibility, the guideline working group recommends using active maximum retrusion as the reference point for determining the sagittal dimension of the starting MAD position [26, 27]. From the patient’s perspective, the MAD starting position should be.

- comfortable (pain- and tension-free) and
- approximately 50% of maximum protrusion, if feasible (measured from active maximum retrusion to active maximum protrusion in the supine position, as the best of three attempts, in accordance with the DC/TMD criteria [16]).

The horizontal dimension of the starting position of MAD therapy (transverse axis) should take the patient-specific lateral deviation of the mandible that occurs during advancement into account and should be comfortable for the patient (Table 4).

**Table 4:** Recommendations of the guideline development group: maxillomandibular relationship, starting point, and MAD type selection

Recommendation 2	
a) The maxillomandibular relationship (MMR) for the starting position of MAD therapy should be recorded by a dentist with advanced training in sleep medicine Voting results: 8/0/0 (Yes/No/Abstain)	Strong consensus
b) Instruments that allow for the adjustment of jaw position in all three dimensions in a lying position should be used in order to obtain reproducible, painless, and tension-free maxillomandibular relationship records of the MAD starting position Voting results: 7/0/1 (Yes/No/Abstain)	Strong consensus
c) Regarding MAD type selection, any oral appliance considered for MAD therapy should be a custom, adjustable, bimaxillary retained splint system fabricated in a dental laboratory based on dental impressions and a maxillomandibular relationship record, and equipped with protrusion adjustment elements Voting results: 7/0/1 (Yes/No/Abstain)	Strong consensus
a) Statement: Good management of side effects of MAD therapy is essential for achieving good adherence and clinical efficacy of treatment Voting results: 8/0/0 (Yes/No/Abstain)	Strong consensus
b) The MAD should be adjustable in small increments of up to 1 mm and should stably maintain the therapeutic position established by titration Voting results: 7/0/1 (Yes/No/Abstain)	Strong consensus

#### Maxillomandibular relation registration instruments

In order to obtain a reproducible maxillomandibular relation record for the starting position of OAT, dentists should use appropriate registration instruments (bite gauge). The guideline working group recommends the use of registration instruments that permit reproducible adjustment in the three spatial dimensions in a lying and relaxed treatment position, making it easier to obtain reproducible maximum active protrusion and retrusion determinations and painless and tension-free maxillomandibular relationship records.

Different technical features of MAD serve to advance the mandible to a more forward position and, thus, expand the upper airway. There are two basic MAD design types: non-adjustable one-piece (monobloc) appliances and adjustable two-piece (bibloc) appliances. Based on the current scientific evidence, adjustable bibloc MADs with specific features should be used in adults [19, 28–32] (Table 4).

#### MAD type selection and design instructions

Recent research certifies positive treatment outcomes for titratable and non-titratable MADs regarding sleep parameters in patients with mild to moderate OSA [33]. Due to clinical features like adjustability, comfort, design, any oral appliance considered for long-term OAT must be a custom made, adjustable, bimaxillary retained, articulated splint system equipped with adjustable protrusive elements fabricated in a dental laboratory based on dental impressions or scans and a maxillomandibular relationship record, and three-dimensional registrations of the starting position taken with a bite gauge. The MAD should allow for anterior and posterior readjustment of the mandible from the starting position because sagittal adjustment of mandibular position is a key variable determining the effectiveness of MADs in the treatment of OSA and snoring. This sagittal adjustment should be possible in reproducible increments of up to 1 mm. It should permit at least 5-mm forward and 1-mm backward readjustment in increments with adjustment mechanism on the frontal or each lateral side, respectively [19]. For extended durability and good tolerance, the MAD should be made of stable, biocompatible materials with a secondary interlocking effect. Finally, the MAD should be easy to insert and to remove by the patients as well as their caregivers and healthcare providers, as needed [32] (Table 4).

#### Appliance delivery, use, and care

Appliance delivery involves a number of steps required not only in order to ensure the effectiveness of OAT, the longevity of the MAD appliance, and patient adherence, but also in order to minimize the potential side effects of OAT. Integrity and technical design quality of the MAD have to be controlled by the dentist before initial insertion. Next, the appliance is inserted in the patient’s mouth and checked for fit, retention, and lip closure. [34, 35].

The patient must be informed and instructed about the protocol for MAD titration and adjustment. Patients with a diagnosis of OSA must be informed in advance about the need for a sleep medicine physician to assess the treatment effect of the MAD on completion of the titration phase.

Patients must be informed about potential side effects before the start of OAT and also during the course of treatment, if necessary. Jaw exercises must be demonstrated to and practiced with the patient in order to prevent or reduce pain in the jaw muscle and temporomandibular joint areas as well as to ensure the complete reposition of the mandible to its habitual position after removal of the MAD and, thus, to increase patient adherence [36–38] (Table 5).

**Table 5:** Recommendations of the guideline development group: Appliance delivery, use and care instructions

Recommendation 3	
The patient must be given information and instructions on MAD handling, use and care, jaw exercises, and dental recall during the delivery appointment Voting results: 8/0/0 (Yes/No/Abstain)	Strong consensus

#### MAD titration, dental and medical follow-up

Adjustment of a mandibular advancement device during the initial OAT phase is defined as *titration* of the MAD, in accordance with dental sleep medicine requirements. The titration phase starts after a successful acclimatization phase; it consists of adjusting the degree of mandibular advancement (titration, in increments of ≤ 1 mm) and, if necessary in order to reduce side effects, also a horizontal and or vertical adjustment. The aim of these adjustments is to optimize the MAD effect while minimizing the side effects of OAT, which leads to better adherence to the treatment. As the degree of mandibular advancement increases, increased effectiveness of treatment becomes more likely, but there is no linear relationship between the degree of mandibular advancement/readjustment and the effectiveness of MAD therapy [34, 39]. Data on symptoms and clinical measures gathered in outpatient protocols (such as sleep quality, daytime sleepiness, and under certain circumstances home-used sensors monitoring oxygen saturation and heart rate) serve as initial sleep medicine-based target variables for MAD adjustment/titration. The assessment of sleep medical requirements for MAD adjustment/titration should be made after mutual consultation between the referring physician and the dentist. The dentist alone is responsible for performing MAD adjustment/titration. The most important symptom-related outcome measures include snoring and daytime sleepiness, with evaluation via analog scoring or questionnaires, such as the Epworth sleepiness scale (ESS) [3, 40]. Metrological target parameters are derived by evaluating single- and multi-channel polygraphy recordings measuring oxygen saturation, peripheral arterial tone, respiratory airflow, heart rate and body position.

The titration phase ends when the therapeutic position of the MAD has been reached. At this phase, no further adjustment of mandibular position is needed, the patient is satisfied with the result, and the sleep physician has demonstrated that the MAD has a sufficient effect during sleep (sufficient response to MAD therapy) usually after polysomnographic recordings. The MAD should be worn during the entire duration of sleep. As standard procedure, the first dental recall appointment (check-up) should be scheduled 6 months after the end of the titration phase, then annually. Recall intervals can be adjusted in accordance with the individual risk profile of the patient [13] (Table 6).

**Table 6:** Recommendations of the guideline development group: MAD titration, dental and medical follow-up

Recommendation 4	
a) Readjustment of mandibular position in the titration phase of MAD therapy should be achievable in the smallest possible increments of up to 1 mm in order to influence the factors’ efficacy, side effects, and adherence in the best possible manner Voting results: 7/0/1 (Yes/No/Abstain)	Strong consensus
b) At the end of the titration phase, the effectiveness of the therapeutic position of the MAD should be evaluated and confirmed by the sleep physician Voting results: 8/0/0 (Yes/No/Abstain)	Strong consensus

#### Outcome orientation

Assessment of the outcome of MAD therapy includes an evaluation of patient-related aspects such as efficacy, manageability, comfort, side effects, and patient adherence to the treatment. Technical aspects of the assessment of OAT quality are related to the MAD: these are fit, adjustability and titratability, durability, convertibility/repairability, cleanability/hygienics and material biocompatibility (Table 7).

**Table 7:** Recommendations of the guideline development group: outcome orientation

Recommendation 5	
a) Patients must be informed about the potential side effects of MAD therapy and about possible measures for their reduction or prevention before the start of treatment Voting results: 8/0/0 (Yes/No/Abstain)	Strong consensus
b) Statement: Good management of side effects of MAD therapy is essential for achieving good adherence and clinical efficacy of treatment Voting results: 8/0/0 (Yes/No/Abstain)	Strong consensus
c) The MAD should be adjustable in small increments of up to 1 mm and should stably maintain the therapeutic position established by titration Voting results: 7/0/1 (Yes/No/Abstain)	Strong consensus

## Conclusion

The risks and benefits of MAD therapy need to be evaluated from medical and dental perspectives in using an interdisciplinary approach. MAD providers should have sound knowledge about specific appliances and their therapeutic application. MADs should be designed for long-term therapy and must be a custom made, adjustable, bimaxillary retained two-splint system with adjustable protrusive elements, fabricated in a dental laboratory according to dental impressions or scans and three-dimensional registrations of the starting position taken with bite gauge. Explanation of potential side effects of MAD should be given before the start of treatment and instructions on MAD handling during the delivery appointment. Finally, the effectiveness of the therapeutic position of the MAD should be evaluated and confirmed by the sleep physician.

## Data Availability

Not applicable.
